# Factors associated with self-assessed increase in tobacco consumption among over-indebted individuals in Germany: a cross-sectional study

**DOI:** 10.1186/1747-597X-8-12

**Published:** 2013-03-13

**Authors:** Heiko Rueger, Heide Weishaar, Elke B Ochsmann, Stephan Letzel, Eva Muenster

**Affiliations:** 1 Institute for Occupational, Social, and Environmental Medicine, University Medical Center of the University of Mainz, Obere Zahlbacher Strasse 67, Mainz D-55131, Germany; 2 Centre for Population Health Sciences, University of Edinburgh, Teviot Place, Edinburgh EH8 9AG, UK; 3 Institute for Occupational and Social Medicine; Medical Faculty, RWTH Aachen University, Pauwelstrasse 30, Aachen D-52074, Germany

**Keywords:** Over-indebtedness, Bankruptcy, Smoking, Financial stress, Social support

## Abstract

**Background:**

Over-indebtedness is an increasing phenomenon in industrialised nations causing individual hardship and societal problems. Nonetheless, few studies have explored smoking among over-indebted individuals.

**Methods:**

A cross-sectional survey (n=949) on retrospectively assessed changes in tobacco consumption was carried out in 2006 and 2007 among clients of 84 officially approved debt and insolvency counselling centres in Germany (response rate 39.7%). Logistic regressions were performed to explore factors associated with reports of increased smoking after onset of over-indebtedness.

**Results:**

63% of all respondents stated daily or occasional tobacco consumption. Almost one fifth reported an increase in smoking after becoming over-indebted. Females were less likely to report increased smoking than men (aOR 0.66, 95% CI 0.44-0.99) whereas respondents who had been over-indebted for more than 10 years were more likely to report increased smoking than those who had been over-indebted for less than five years (aOR 1.66; 95%-CI 1.00-2.76). The odds of increased smoking were also elevated among those who reported that their families and friends had withdrawn from them as a consequence of their over-indebtedness (aOR 1.82; 95%-CI 1.06-3.14).

**Conclusions:**

The study identifies over-indebted individuals and particularly over-indebted men as a high-risk group of smokers. Low levels of social embeddedness/support were associated with a further increase in smoking after becoming over-indebted. Given recent increases of over-indebtedness, the findings highlight the need to develop appropriate public health policies.

## Background

The current financial and economic crisis has resulted in several European countries having stagnating economic growth, increasing unemployment rates and marked reductions in public welfare spending. Possible health consequences, including increased suicide rates, problematic health behaviour and mental disorders, are beginning to receive some (public and) academic attention [1-3]. Given that research shows that loss of employment and low income are major drivers of over-indebtedness [4], it can be assumed that the current crisis will lead to an increase in over-indebtedness. Over-indebtedness of individuals or households describes the situation when, over an extended period, a household’s income is insufficient to service its debts on time despite a reduction of the standard of living [5]. Over the last decades, the number of over-indebted private households and individuals has increased. Current estimates suggest that approximately three million German households are affected and reveal a high share of over-indebted households in many other industrialised nations, varying between 2% in Finland and 12% in the United States [6,7]. Over-indebtedness and financial difficulties are related concepts as they usually affect individuals of lower socio-economic status. Recent studies have found an association with health and health behaviour [8-13], revealing higher prevalences of overweight and obesity [14], back pain [15] and mental disorder [16] in over-indebted individuals in Germany. Research in high-income countries has further suggested an association between financial difficulties and smoking cessation, with those experiencing financial stress being less likely to make quit attempts or successfully quit smoking [17-20]. The study at hand adds to this area of research and examines smoking in over-indebted individuals in Germany and factors associated with changes in tobacco consumption in times of over-indebtedness.

## Methods

### Study design and participants

The study utilized data from the “OI-survey”, a cross-sectional survey on health status and health-related lifestyles in over-indebted individuals using a self-administered German language questionnaire. 949 eligible individuals who attended one of all 84 officially approved debt and insolvency counselling centres in the German Länder (provinces) of Rhineland-Palatinate (n=666) and Mecklenburg-West Pomerania (n=283) and satisfied the recruitment criteria (attended a minimum of two counselling sessions at one of the centres between 2006 and 2007, ≥16 years of age) were enrolled in the study. In order to obtain a sample of unrelated individuals, only one individual per household was interviewed. If more than one individual attended the counselling session, the over-indebted person was interviewed. If both attendees (usually married or unmarried couples) were affected, it was left to the attendees to decide who would complete the questionnaire. The respondent who filled in the questionnaire was asked to report on his or her own smoking habits. The response rate was 39.7%. All 84 officially approved debt and insolvency counselling centres located in the two provinces were included in the survey. In Germany, debt and insolvency counselling offered by officially approved counselling centres is free of charge for the attendee. During the first appointment, the counselor assesses whether the attendee meets the eligibility criteria for receiving debt and insolvency counselling of “being in serious financial difficulties”. The fact that demand for debt counselling exceeds supply by far and the rigorous recruitment criteria of the study (participants had to have attended at least two counselling sessions) meant that study participants were almost certainly over-indebted. Further study design details are published elsewhere [11,14,15]. A test for representativeness conducted among the Rhineland-Palatinate sample reported no differences in sex and age compared to official 2006 statistics of all counselling clients. Marital status, nationality and amount of debt, however, revealed statistically significant differences, with non-German citizens and married individuals being slightly underrepresented and single individuals being slightly overrepresented.

Ethical approval was obtained from the medical association of Rhineland-Palatinate, Germany.

### Measurements

#### Outcome measures

An individual’s smoking status was surveyed using the item “Have you smoked previously or do you currently smoke?” Participants who reported being daily or occasional smokers were classified as smokers. Participants who reported that they used to smoke were classified as ex-smokers. Changes in smoking after onset of over-indebtedness were examined using the item “Has your lifestyle regarding consumption of tobacco products changed since you have become over-indebted?” Possible answers were “I consume less tobacco than before”, “I consume the same amount of tobacco as before”, “I consume more tobacco than before” and “Does not apply”. The increase in tobacco consumption after onset of over-indebtedness was chosen as the target category (=1), whereas participants who reported decreased or unchanged tobacco consumption after onset of over-indebtedness served as the reference category (=0).

#### Covariates

In order to measure changes regarding social embeddedness since the beginning of over-indebtedness, participants were asked whether anything had changed in their family or circle of acquaintances since they started suffering from financial hardship. An index including the following three categories was generated: “neither family members nor friends have withdrawn”, “either family members or friends have withdrawn”, and “family members and friends have withdrawn”. The measurement of perceived social support was carried out using the standard F-SozU, a measuring instrument based on self-assessment of perceived social support. A short version of the scale with 14 items (Cronbach’s Alpha=.94) was used [21]. The resulting scale was stratified *a priori* into four categories. Health awareness was surveyed by asking respondents about the degree to which they were concerned about their health with the first and last two attributes of a five point categorical scale being subsumed. The duration of over-indebtedness was considered in order to measure chronic stress and categorised *a priori* into “five years or less”, “six to ten years” and “more than ten years”. The question “To what degree do you feel burdened by your debts?” (response categories: “not at all”, “slightly”, “moderately”, “highly” and “very highly”) was used as an additional proxy for an individual’s perceived levels of stress.

Well-established socio-demographic confounders including age, gender, formal education, net household income and marital status were categorised *a priori*. The categorisation of these measures is detailed in Table 1.

**Table 1:** Descriptive statistics of individuals who answered the question regarding a possible change in tobacco consumption after onset of over-indebtedness (n=572)

	**N**	**%**
Sex		
*Male*	294	51.4
*Female*	278	48.6
Age		
*18-30*	131	22.9
*31-40*	157	27.4
*41-50*	189	33.0
*51+*	92	16.1
*n.a.*	3	0.5
Educational level		
*≤ 9 years*	380	66.4
*> 9 years*	192	33.6
Income		
*≤ 1500 €*	445	77.8
*> 1500 €*	127	22.2
Marital status		
*Married*	156	27.3
*Unmarried*	193	33.7
*Divorced/Separated/Widowed*	219	38.3
*n.a.*	4	0.7
Duration of over-indebtedness		
*≤ 5 years*	201	35.1
*6 to 10 years*	186	32.5
*> 10 years*	166	29.0
*n.a.*	19	3.3
Social support		
*High*	129	22.6
*Less than high*	218	38.1
*More than low*	139	24.3
*Low*	79	13.8
*n.a.*	7	1.2
Health awareness		
*High*	203	35.5
*Medium*	265	46.3
*Low*	100	17.5
*n.a.*	4	0.7
Changes in social embeddedness since onset of over-indebtedness		
*No withdrawal*	303	53.0
*Friends or relatives withdrawn*	155	27.1
*Friends and relatives withdrawn*	103	18.0
*n.a.*	11	1.9

Notes: OI-survey.

### Statistical methods

Initially, smoking in over-indebted individuals and overall changes in tobacco consumption (i.e. increased, decreased or unchanged consumption) following onset of over-indebtedness were examined. For this, the analysis covered the complete sample of 949 participants. In a second step, factors associated with an increase in tobacco consumption after onset of over-indebtedness were examined. For this analysis, 572 current and former smokers who answered the question regarding a possible change in smoking were investigated.

Smoking prevalence and changes in prevalence after onset of over-indebtedness were calculated. To identify potential risk factors for an increase in smoking, multivariate binary logistic regressions were performed (Table 2). In a first step (model 1), perceived social support was analysed in combination with the covariates and confounders mentioned above. In a second step (model 2), changes in social embeddedness following onset of over-indebtedness was additionally included in the analysis. Adjusted odds ratios and 95%-confidence intervals were calculated, with the level of statistical significance being set at α≤0.05. Missing data were excluded for bivariate analyses and placed in a separate category for multivariate analyses.

**Table 2:** Factors associated with increased smoking in over-indebted individuals (reference category: decreased or unchanged tobacco consumption)

	***Increase in smoking***	***Model 1***^***c***^		***Model 2***^***cd***^	
	**Row percentages**	**aOR**	**95% CI**	**aOR**	**95% CI**
Sex	*p=.018, df=1*^*a*^				
*Male (ref.)*	35.0	1		1	
*Female*	25.9	0.66	0.44-0.99	0.67	0.45-0.99
Age	*p=.393, df=3*^*a*^				
*18-30 (ref.)*	26.7	1		1	
*31-40*	35.0	1.03	0.57-1.84	1.00	0.56-1.80
*41-50*	31.7	0.80	0.45-1.44	0.78	0.43-1.42
*51+*	27.2	0.63	0.30-1.30	0.62	0.30-1.29
Educational level	*p=.665, df=570*^*b*^				
*≤ 9 years (ref.)*	30.0	1		1	
*> 9 years*	31.8	1.08	0.73-1.62	1.12	0.75-1.68
Income	*p=.119, df=570*^*b*^				
*≤ 1500 € (ref.)*	29.0	1		1	
*> 1500 €*	36.2	1.34	0.84-2.12	1.38	0.86-2.20
Marital status	*p=.251, df=2*^*a*^				
*Married (ref.)*	32.1	1		1	
*Unmarried*	26.4	0.73	0.42-1.26	0.78	0.45-1.36
*Divorced/Separated/Widowed*	33.8	1.10	0.68-1.77	1.14	0.70-1.86
Duration of over-indebtedness	*p=.010, df=551*^*b*^				
*≤ 5 years (ref.)*	26.4	1		1	
*6 to 10 years*	29.0	1.05	0.65-1.68	1.04	0.64-1.68
*> 10 years*	39.2	1.76	1.00-2.73	1.66	1.00-2.76
Social support	*p<.001, df=563*^*b*^				
*High (ref.)*	22.5	1		1	
*Less than high*	26.6	1.21	0.71-2.06	1.10	0.64-1.89
*More than low*	36.7	1.88	1.07-3.30	1.51	0.83-2.74
*Low*	45.6	2.78	1.45-5.26	1.99	0.99-3.98
Health awareness	*p<.001, df=566*^*b*^				
*High (ref.)*	22.2	1		1	
*Medium*	33.2	1.59	1.03-2.47	1.66	1.07-2.58
*Low*	42.0	1.98	1.15-3.43	2.15	1.23-3.76
Changes in social embeddedness since onset of over-indebtedness	*p<.001, df=559*^*b*^				
*No withdrawal (ref.)*	24.1			1	
*Friends or relatives withdrawn*	35.5			1.45	0.91-2.31
*Friends and relatives withdrawn*	41.7			1.82	1.06-3.14

Notes: OI-survey; ^a^ chi^2^ test; ^b^ t-test (Spearman correlation); ^c^ multivariate binary logistic regression (n=572); ^d^ changes in social embeddedness following onset of over-indebtedness was additionally included in the analysis.

SPSS version 17 (SPSS Inc., Chicago, Illinois) statistical software package was used for all analyses.

## Results

### Smoking in over-indebted individuals

A total of 598 over-indebted individuals (63.0%) reported to either smoke daily or occasionally. Men were significantly more likely to smoke (67.7%) than women (59.3%, χ^2^=7.142, df=1, p=.008). 168 participants (17.7%) reported to be ex-smokers. 74.7% (n=572) of all current and former smokers answered the question regarding a possible change in tobacco consumption after onset of over-indebtedness. 39.0% of this subpopulation (n=223, 23.5% of the entire study sample) reported to smoke less, 30.4% (n=174, 18.3% of the entire study sample) reported no changes in tobacco consumption, and 30.6% (n=175, 18.4% of the entire study sample) reported increased smoking.

### Description of the study sample

The characteristics of the subpopulation of over-indebted individuals who reported changes in tobacco consumption after onset of over-indebtedness examined in this study (N=572) are presented in Table 1.

About one half of the study sample was female (48.6%) and aged under 40 years (50.3%), with age ranging from 18 to 79 years. About one third was divorced, separated or widowed (38.3%) and had undergone more than nine years of schooling (33.6%). One fifth had a net household income of more than 1500 Euros (22.2%). A third of the study sample had been over-indebted for ten years or more (29.0%), 35.5% reported high levels of health awareness, and 22.6% reported high levels of social support. The withdrawal of friends and relatives since onset of over-indebtedness was reported by a fifth of all participants (18.0%), whereas more than one fourth reported that either friends or relatives had withdrawn (27.1%) and about half had experienced no withdrawal (53.0%).

### Factors associated with increased smoking

According to the bivariate analysis, sex, duration of over-indebtedness, health awareness, perceived social support and changes in social embeddedness since onset of over-indebtedness were significantly associated with the outcome measure of increased smoking (Table 2).

Men had a significantly higher risk to increase tobacco consumption during over-indebtedness than women. 35.0% of the men as opposed to 25.9% of the women reported such behavioural change. This effect was maintained when multivariate analysis was applied to the data (Table 2). Other socio-demographic and socio-economic variables, including age, education, income and marital status, showed no significant effects in bivariate or multivariate analyses.

The measure for perceived stress revealed high levels of stress among over-indebted individuals and almost no variation: 87% of the study sample reported feeling highly or very highly burdened, suggesting that over-indebtedness placed an extraordinary strain on the affected individuals. Accordingly, the analysis presented in Table 2 revealed that the longer the period of over-indebtedness, the more likely individuals were to increase tobacco consumption. 39.2% of all respondents who had been over-indebted for more than 10 years reported an increase in smoking, whereas only 26.4% of those who had experienced over-indebtedness for less than 5 years reported the same. This difference could also be seen in the fully adjusted model 2 (aOR 1.66; 95%-CI 1.00-2.76). The hypothesis that duration of over-indebtedness has a significant impact on changes in smoking could thus be confirmed.

Individuals with higher levels of health awareness had a lower risk of increased tobacco consumption after onset of over-indebtedness. 42.0% of all participants who reported low levels of health awareness reported increased smoking, whereas this was the case for only 22.2% of all participants reporting high levels of health awareness. This finding was also depicted in the multivariate analysis and thereby proved to be independent of other factors investigated (Table 2, model 2).

Withdrawal of friends and relatives was correlated with a significantly higher risk of increased smoking. Respondents who reported that friends and relatives withdrew from them after onset of over-indebtedness had a 1.82 times higher risk of increased smoking in the fully adjusted model (95%-CI 1.06-3.14) compared to participants who did not report that their friends and relatives withdrew (Table 2, model 2). As expected, changes in social embeddedness were closely related to perceived social support (Spearman correlation=-.412, t=10.657, df=557, p<.001). Moreover, increased tobacco consumption was observed in those who experienced lower levels of social support. Percentages of increased smoking varied between 22.5% in participants reporting strong and 45.6% in those reporting little social support (Table 2). This effect remained significant in model 1 but lost statistical significance when controlling for changes in social embeddedness after onset of over-indebtedness (model 2), reflecting the close relationship between both variables. It is possible that previous changes in social embeddedness had an impact on the reporting of perceived social support at the time of the survey.

## Discussion

Considering that approximately 16 million people smoke and 3 million households are estimated to be affected by over-indebtedness in Germany alone, the absence of studies which have investigated the relationship between smoking and an individual’s financial strain as experienced in a situation of over-indebtedness is staggering. On the basis of a cross-sectional survey conducted among over-indebted individuals in Germany, this study investigates smoking prevalence and factors associated with increased tobacco consumption in over-indebted individuals.

A striking result is the high smoking prevalence among over-indebted individuals, with 63% of all respondents reporting daily or occasional tobacco consumption. In comparison, smoking prevalence in the general German population is considerably lower (34%) and even the subpopulation with the highest tendency to smoke (40- to 59-year-old men of low socio-economic status) are significantly less likely to smoke (54.8%) than over-indebted individuals [22]. The findings suggest that smoking is particularly prevalent among over-indebted individuals.

In addition, smokers seem to change their consumption in times of financial hardship with one third reporting an increase in tobacco consumption after onset of over-indebtedness. Health awareness, social embeddedness and perceived social support (in case of not controlling for social embeddedness) were associated with lower risk of increased tobacco consumption whereas duration of over-indebtedness was associated with higher risk. In line with other research showing higher smoking prevalence among men, male respondents were more likely to smoke and had a significantly higher risk to increase their tobacco consumption during over-indebtedness than women.

A number of limitations of the study have to be considered when discussing the data. First, the cross-sectional design which does not allow for causal explanations regarding over-indebtedness, prevalence and changes in smoking constitutes one of the major weaknesses of our study. Participants’ responses regarding changes since onset of over-indebtedness serve as proxies to assess changes over time, but a potential recall bias due to retrospective smoking behavior measures [23] has to be acknowledged. Moreover, the respective questionnaire item directly linked financial problems to possible changes in smoking patterns and might therefore have provoked anticipated answers. The fact that almost one third of all respondents reported no changes in smoking, however, suggests that respondents did not seem to feel obliged to report changes into any specific direction. Second, all results are based on self-reports. While previous studies suggest that self-reports of smoking provide valid data [24], the reliance on self-reports for all measures may imply limitations for the interpretation of our data. Third, a potential selection bias due to a low response rate and sampling having to be based on consent might have skewed the results. It needs to be noted, however, that low response rates can be expected when conducting research on those of low socio-economic status [25].

One of the difficulties in understanding and tackling the problem of smoking in over-indebted individuals is the lack of knowledge about potential pathways between the two factors. In addition to previous research discussing increased stress, lack of social support, and lower rates of successful quit attempts as potential mediators of the correlation between low socio-economic status and smoking [26-28], our study points to a number of factors which might explain why many of over-indebted individuals increase their smoking.

As expected, health awareness is associated with a lower risk of increased tobacco consumption, suggesting that individuals who pay more attention to their health are more likely to be concerned about their tobacco consumption. The duration of over-indebtedness seems to be a crucial factor when analysing changes in tobacco consumption. One possible explanation for the fact that increased tobacco consumption correlates with duration of over-indebtedness might be that increased tobacco consumption is employed as a mechanism to cope with the financial, emotional and social stress caused by over-indebtedness. This hypothesis is supported by previous research which suggests that disadvantaged smokers perceive smoking as a strategy to deal with daily stress [28-30]. Almost all respondents in our study reported to be burdened by their debts, suggesting higher levels of stress among this population and potentially higher urge to develop coping mechanisms to deal with the situation. On the other hand, the study highlights the role of social embeddedness and support for understanding why over-indebted individuals are particularly likely to smoke and increase their tobacco consumption after onset of over-indebtedness. The analysis shows that the risk of increased tobacco consumption is higher among individuals who experience lower levels of social embeddedness and social support. Social embeddedness and social support seem to be crucial factors for coping with financial and emotional strain and constitute protective factors regarding tobacco consumption. Assuming that an individual’s perceived social support correlates with his or her embeddedness in a social structure, the progressive withdrawal of family members and friends might lead to lower levels of perceived social support in over-indebted individuals. As perceived social support decreases, an increase in tobacco consumption can be expected. Although the inverse relationship between social embeddedness/perceived social support and tobacco consumption supports previous research which identifies social support as a protective factor when coping with stress [31], the considerable research gap about social embeddedness/social support and changes in tobacco consumption and the fact that our findings are based on self-reports highlights the need for further research in this specific area.

## Conclusions

Our analysis emphasises the need for health promotion interventions to be tailored to the social context in which over-indebted people live and for policy change to promote health among this group of high-risk individuals. In order to develop their full potential, attempts to decrease smoking prevalence should not only focus on changing individual behaviour but need to incorporate policies which address the social determinants of health. While population-wide tobacco control interventions can reduce the social gradient, targeted interventions are needed to reach particular high-risk groups, including individuals who are burdened by financial strain [32]. In this context, our study shows that over-indebted individuals and particularly over-indebted men constitute a specific target group for health promotion. The fact that almost one fourth of all over-indebted individuals reported that they had managed to reduce smoking since the onset of over-indebtedness suggests that considerable potential exists among this population for smoking reduction and cessation measures, a finding which should be an encouragement when developing smoking cessation services. This study identifies over-indebted individuals as a high-risk group for detrimental health behaviour and thus corresponds with research relating over-indebtedness and financial difficulties to a number of other health-related behaviours [10,12-15]. We acknowledge that the size of the effects measured in this study is relatively weak. However, the fact that approximately 6 million people are estimated to be affected by over-indebtedness in Germany alone illustrates the magnitude of the problem. Given the anticipated number of affected individuals, even small effects can be assumed to have a large impact on overall public health. The findings thus justify suggestions for policy and practice.

One of the policy implications of this study concerns the co-ordination between sectors which have access to financially burdened individuals. A 2009 UK article, which draws attention to the correlation between debt and mental health, suggests “co-ordinated ‘debt care pathways’ between local health and advice services” [33]. Similarly, the findings of this study indicate that a coordinated response is needed to tackle the relationship between debt and smoking. Policies are needed to ensure that debt counsellors as well as medical professionals are alert to the problem, able to assess the prevalence of smoking among their clients and act appropriately. A response to the problem should include respective training for people working in the field, the development of routine assessments of risky health behaviour among over-indebted individuals and the systematic coordination between debt counselling centres and the health service. Debt counselling centres should further be considered as an appropriate, and perhaps not fully exploited, setting to implement intensified and target-specific health promotion interventions. Our analysis suggests that the provision of smoking cessation and other health promotion measures alongside debt counselling promises to have a considerable potential for reaching a high-risk group of heavy smokers and could constitute a highly effective health promotion measure.

## Competing interests

This work was supported by the German Federal Ministry for Science, Further Education, Research and Culture of Rhineland-Palatinate as part of the research cluster “Corporate interdependence and social networks”. The funding organization was not involved in any stage at this research.

No non-financial competing interests declared.

## Authors’ contributions

HR analysed the data and drafted and revised the paper. He is guarantor. HW drafted and revised the paper. EBO revised the paper. SL initiated the study and revised the paper. EM initiated the study, designed data collection tools, monitored data collection, and revised the paper. All authors read and approved the final manuscript.
